# Enhancing photocatalytic H_2_O_2_ production with Au co-catalysts through electronic structure modification

**DOI:** 10.1038/s41467-024-47624-7

**Published:** 2024-04-13

**Authors:** Xidong Zhang, Duoduo Gao, Bicheng Zhu, Bei Cheng, Jiaguo Yu, Huogen Yu

**Affiliations:** 1 Laboratory of Solar Fuel, Faculty of Materials Science and Chemistry, China University of Geosciences, 68 Jincheng Street, Wuhan, P. R. China; 2 State Key Laboratory of Silicate Materials for Architectures, Wuhan University of Technology, 122 Luoshi Road, Wuhan, P. R. China; 3 State Key Laboratory of Advanced Technology for Material Synthesis and Processing, Wuhan University of Technology, Wuhan, P. R. China

**Keywords:** Photocatalysis, Photocatalysis

## Abstract

Gold-based co-catalysts are a promising class of materials with potential applications in photocatalytic H_2_O_2_ production. However, current approaches with Au co-catalysts show limited H_2_O_2_ production due to intrinsically weak O_2_ adsorption at the Au site. We report an approach to strengthen O_2_ adsorption at Au sites, and to improve H_2_O_2_ production, through the formation of electron-deficient Au^δ+^ sites by modifying the electronic structure. In this case, we report the synthesis of TiO_2_/MoS_x_-Au, following selective deposition of Au onto a MoS_x_ surface which is then further anchored onto TiO_2_. We further show that the catalyst achieves a significantly increased H_2_O_2_ production rate of 30.44 mmol g^−1^ h^−1^ in O_2_-saturated solution containing ethanol. Density functional theory calculations and X-ray photoelectron spectroscopy analysis reveal that the MoS_x_ mediator induces the formation of electron-deficient Au^δ+^ sites thereby decreasing the antibonding-orbital occupancy of Au-O_ads_ and subsequently enhancing O_2_ adsorption. This strategy may be useful for rationally designing the electronic structure of catalyst surfaces to facilitate artificial photosynthesis.

## Abstract (web summary)

Photocatalytic H_2_O_2_ production using Au is hindered by its inherently weak O_2_ adsorption. Herein, the authors modify the electronic structure of Au with MoS_x_ to form electron deficient Au sites to promote O_2_ adsorption and H_2_O_2_ production.

## Introduction

Solar-driven photocatalytic H_2_O_2_ production through oxygen reduction reaction (ORR) is a promising method for addressing energy and environmental crises due to its low energy consumption, safety, and environmental friendliness^1–5^. However, the production of H_2_O_2_ via photocatalysts is hindered by the low efficiency of electron transfer and interface reaction^6–8^, resulting in suboptimal H_2_O_2_ yields^9,10^. To address these challenges, cocatalyst deposition on the surface of photocatalysts can not only effectively promote electron transfer but also provide specialized active sites to facilitate interfacial ORR^11–13^. It is well known that photocatalytic H_2_O_2_ production via ORR on the active sites of cocatalysts involves multiple fundamental steps, such as O_2_ adsorption, intermediate *OOH formation, and H_2_O_2_ desorption^8,14,15^. Of these, O_2_ adsorption at active sites is one of the most important processes, as it facilitates the formation of intermediate *OOH and its further conversion into H_2_O_2_^16–19^. Sabatier principle^20^. Suggests that the interaction between active sites and adsorbates must have an optimal binding energy. Further research indicates that the electron configuration of active sites fundamentally determines their interaction with adsorbates, influencing their adsorption/desorption performances^21^. However, in photocatalytic H_2_O_2_ production, current cocatalysts usually suffer from a mismatch in the electronic configuration between the active site and the adsorbed O_2_, leading to either excessively strong or weak O_2_ adsorption, which in turn limits H_2_O_2_-production rates^22–24^. Therefore, it is quite meaningful and challenging to modulate the electronic configuration of cocatalysts and optimize their oxygen adsorption strength to achieve efficient H_2_O_2_ production.

**Fig. 1:**
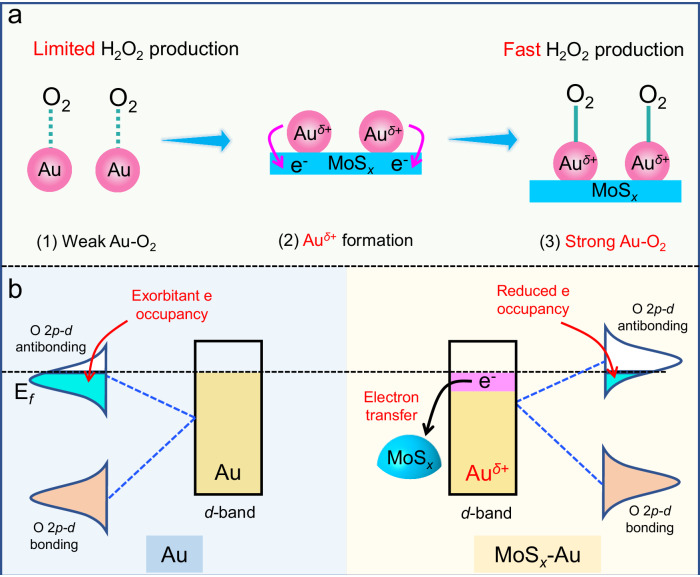
Strategy to design efficient electron-deficient Au^δ+^ cocatalyst for improving H_2_O_2_-production kinetics. **a** Schematic illustration of electron-deficient Au^*δ*+^ formation to reinforce Au-O_ads_ bond: (1) weak Au-O_ads_ bond on Au surface; (2) the formation of electron-deficient Au^*δ*+^ sites by MoS_*x*_ incorporation; (3) strong Au-O_ads_ bond on MoS_*x*_-Au surface. **b** Schematic diagram about reducing the antibonding-orbital occupancy of Au-O_ads_ by the free-electron transfer from Au to MoS_x_ cocatalyst.

Currently, noble metal cocatalysts (Pd, Pt, and Au) have made significant advancements in improving photocatalytic ORR for H_2_O_2_ production^25–28^. Notably, Au cocatalysts usually exhibit a higher photocatalytic H_2_O_2_-production activity, attributed to the effective interfacial charge transfer between photocatalysts and Au nanoparticles^29^. This transfer enables the rapid movement of photogenerated electrons from the photocatalysts to the Au surface, facilitating the reduction of adsorbed O_2_ to H_2_O_2_ through either a two-step single-electron or a one-step two-electron ORR process^30–32^. However, metallic Au usually exhibits weak oxygen adsorption characteristics due to its intrinsic electronic structure (Fig. 1a-(1))^33,34^, which limits the formation of the *OOH intermediate and subsequent H_2_O_2_ production. Consequently, precise modulation of Au’s electronic structure is extremely crucial to optimize O_2_ adsorption and enhance photocatalytic H_2_O_2_ production. For instance, Tsukamoto et al. demonstrated increased electronic density in Au by forming an Au-Ag alloy, reducing H_2_O_2_ decomposition on Au sites^35^. Moreover, Wang et al. prepared an efficient core-shell Cu@Au-modified BiVO_4_ nanostructure. This structure reduces negative charge accumulation at the Au active sites by forming an ohmic contact with Cu/BiVO_4_, thereby enhancing the adsorption of O_2_ and its intermediate *OOH, leading to efficient photocatalytic H_2_O_2_-production performance^36^. Although the introduction of alloy and bimetallic core-shell structures have efficiently enhanced the photocatalytic activity of Au sites, the relationship about the H_2_O_2_-production activity, the Au-O_ads_ bonds, and the electronic structure of Au remains unclear. Fortunately, the molecular orbital theory clearly states that the antibonding-orbital occupancy degree between a metal and its adsorbate usually determines its adsorption energy^37^, which provides a theoretical basis for the modulation of bond strength between Au and O_2_. Inspired by this, selectively decreasing the antibonding-orbital occupancy of Au-O_ads_ is expected to further enhance the O_2_ adsorption on Au, potentially achieving efficient photocatalytic H_2_O_2_ production. However, there has been limited research focusing on this approach to date.

**Fig. 2:**
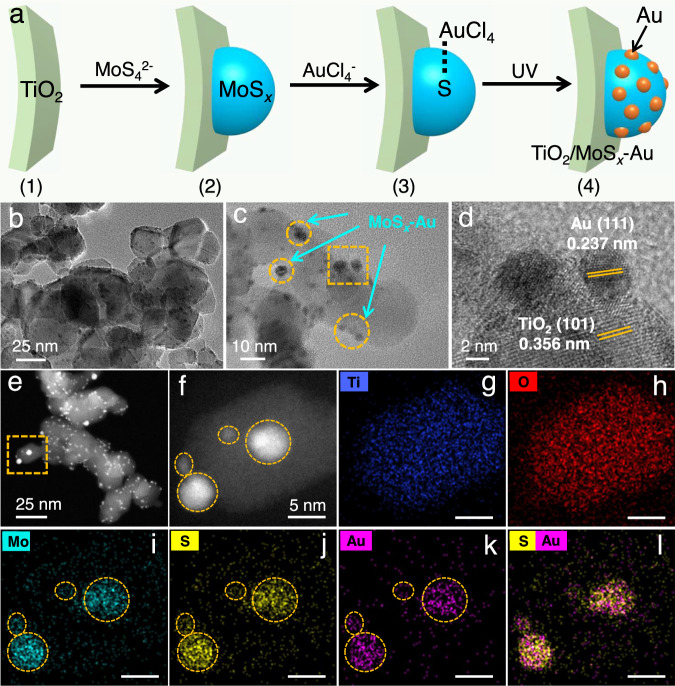
Synthetic strategy and morphology characterization. **a** Schematic illustration for the synthesis of TiO_2_/MoS_*x*_-Au by the initial lactic acid-induced MoS_*x*_ deposition on the TiO_2_ surface and subsequent S-induced selective photodeposition of Au cocatalyst onto the MoS_*x*_ surface. **b**, **c** TEM, **d** HRTEM, **e**, **f** HAADF-STEM, and **g**–**l** elemental mapping pictures of TiO_2_/MoS_*x*_-Au photocatalyst.

In this work, we propose an approach to strengthen the Au-O_ads_ bonds by modifying the electronic structure of Au active site. This is achieved through the introduction of molybdenum sulfide (MoS_*x*_) as an electron mediator to decrease the antibonding-orbital occupancy of Au-O_ads_. In this case, the MoS_*x*_ mediator serves to adjust the electronic structure of Au cocatalyst, resulting in the creation of electron-deficient Au^*δ*+^ active sites and subsequently accelerating H_2_O_2_ production (Fig. 1a-(2) and -(3)). To this end, TiO_2_/MoS_*x*_-Au photocatalyst was synthesized by a two-step method. This process involves the initial MoS_*x*_ deposition onto the TiO_2_ surface and subsequent S-induced selective photodeposition of Au cocatalyst onto the MoS_*x*_ surface. The resulting TiO_2_/MoS_*x*_-Au photocatalyst achieves a boosted H_2_O_2_-production rate of 30.44 mmol g^−1^ h^−1^, which is 25.4 and 1.3 times higher than TiO_2_ and TiO_2_/Au, respectively. Density functional theory (DFT) calculations and ex-situ X-ray photoelectron spectroscopy (XPS) analysis have confirmed the effective reduction of *d*-orbital electron on Au cocatalyst upon the introduction of MoS_*x*_, leading to a decrease in antibonding-orbital occupancy in Au-O_ads_ (Fig. 1b). Consequently, the Au-O_ads_ bonds are significantly reinforced, which in turn enhances the rate of photocatalytic H_2_O_2_ production. This work focuses on modifying the electron structure of Au cocatalyst to reduce the antibonding-orbital occupancy of Au-O_ads_, offering a promising approach to enhance O_2_ adsorption for efficient photocatalytic H_2_O_2_ production.

## Results and discussion

### Synthesis and characterization of TiO_2_/MoS_*x*_-Au

To realize the successful deposition of MoS_*x*_-Au cocatalyst on the surface of TiO_2_ photocatalyst, a facile two-step route was carried out at room temperature (Fig. 2a), including the initial deposition of MoS_*x*_ on the TiO_2_ surface and the subsequently selective photodeposition of Au onto the MoS_*x*_ surface (Supplementary Fig. 1). First, (NH_4_)_2_MoS_4_ solution was mixed with a lactic acid solution to form brown MoS_*x*_ colloidal nanoparticles (Supplementary Fig. 1a-(1)). Subsequently, TiO_2_ nanoparticles were uniformly dispersed into this colloidal solution with constant stirring. The positive charge on the TiO_2_ nanoparticles in the lactic acid solution allowed for the efficient adsorption of MoS_*x*_ colloidal nanoparticles onto the TiO_2_ surface via electrostatic self-assembly (Supplementary Fig. 1a-(2), c). The deposition of MoS_*x*_ on the TiO_2_ surface can be verified by Fourier transform infrared (FTIR) spectroscopy and Raman spectra analyses (Supplementary Fig. 2). A new FTIR peak for S-S vibration and a new Raman peak for Mo-S can be observed, providing strong evidence for the MoS_*x*_ formation^38,39^. With the further addition of HAuCl_4_ solution into the TiO_2_/MoS_*x*_ suspension, AuCl_4_^-^ ions can be selectively adsorbed onto the MoS_*x*_ surface via the strong interaction between S and Au atoms (Fig. 2a)^40^. Upon light irradiation, the AuCl_4_^-^ ions were in situ reduced to form Au nanoparticles on the MoS_*x*_ surface (Supplementary Figs. 1a-(3), d), evident from a color change from light brown to purple (Supplementary Fig. 1b), revealing the successful synthesis of TiO_2_/MoS_*x*_-Au photocatalyst. The above result can further be supported by X-ray diffraction (XRD) and Raman spectra. Compared with the TiO_2_/MoS_*x*_ sample, a new XRD peak of Au at 38.1° and an Au-S Raman peak^41,42^ confirm the selective deposition of Au nanoparticles on the MoS_*x*_ surface (Supplementary Figs. 3 and 4).

Transmission electron microscopy (TEM) analysis was employed to further verify the selective deposition of Au on the MoS_*x*_ surface within the TiO_2_/MoS_*x*_-Au photocatalyst. As depicted in Fig. 2b–d, numerous dark spots on the TiO_2_ surface are observed, which can be attributed to the MoS_*x*_-Au cocatalyst. The high-angle annular dark-field (HAADF) images (Fig. 2e, f) further indicate that the MoS_*x*_-Au nanoparticles were effectively deposited on the TiO_2_ surface. The corresponding energy-dispersive X-ray spectroscopy (EDS) mapping images (Fig. 2g–l) show that four Au nanoparticles exhibit uniform and same distribution with the Mo and S elements on the TiO_2_ particles, unequivocally indicating that all the Au nanoparticles are selectively deposited on the MoS_*x*_ surface via the self-assembly of S-Au. Ultraviolet-visible absorption spectra (UV-Vis) demonstrate a typical surface plasmon resonance (SPR) absorption of Au (ca. 540 nm) (Supplementary Fig. 5)^40,43,44^, suggesting that Au was effectively deposited on the surfaces of both TiO_2_ and TiO_2_/MoS_*x*_. Noticeably, the SPR signal in the TiO_2_/MoS_*x*_-Au appears relatively weaker than that of the TiO_2_/Au, which can be attributed to the strong interaction between Au and S atoms. According to the inductively coupled plasma optical emission spectrometry (ICP-OES) results (Supplementary Table S1), the contents of Mo, S, and Au elements in the TiO_2_/MoS_*x*_-Au photocatalyst are 0.62, 0.8, and 3.34 wt%, respectively, indicating the presence of MoS_*x*_ and Au in the TiO_2_/MoS_*x*_-Au system. These results (HRTEM, UV-Vis, and ICP-OES) collectively support the selective deposition of Au on the MoS_*x*_ surface.

### Photocatalytic performance

**Fig. 3:**
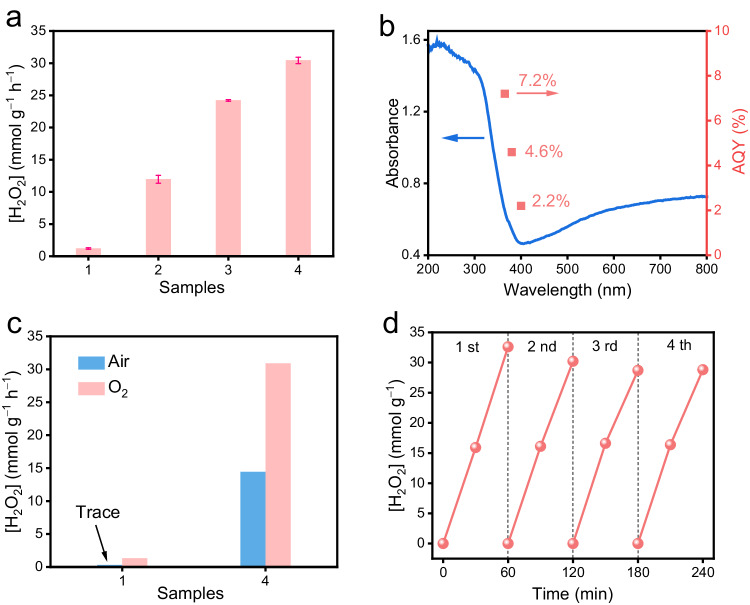
Photocatalytic H_2_O_2_-production activities and stability. **a** Photocatalytic H_2_O_2_-production performance for different samples in an ethanol-water solution (10% vol.): (1) TiO_2_, (2) TiO_2_/MoS_*x*_, (3) TiO_2_/Au, and (4) TiO_2_/MoS_*x*_-Au. The error bars (mean ± standard deviation) were calculated based on three independent photocatalytic experiments. **b** The AQY of H_2_O_2_ production as a function of wavelength on TiO_2_/MoS_*x*_-Au (red dot), and the UV-vis absorbance spectrum (blue curve). **c** The photocatalytic H_2_O_2_-production performance over (1) TiO_2_ and (4) TiO_2_/MoS_*x*_-Au in the O_2_-saturated and air condition. **d** Recycling H_2_O_2_-production performance of TiO_2_/MoS_*x*_-Au.

The photocatalytic activities for H_2_O_2_ production were conducted in an O_2_-saturated ethanol solution under Xe lamp irradiation. As shown in Fig. 3a, TiO_2_ exhibits a low H_2_O_2_-production rate of 1.20 mmol g^−1^ h^−1^. However, the introduction of Au nanoparticles onto the TiO_2_ surface leads to an improved H_2_O_2_-production rate (24.22 mmol g^−1^ h^−1^) for the resulting TiO_2_/Au. With the further incorporation of MoS_*x*_ cocatalyst into TiO_2_/Au, the TiO_2_/MoS_*x*_-Au shows a significant enhancement in the photocatalytic H_2_O_2_-production activity. Further investigation indicated that the H_2_O_2_-production activity of TiO_2_/MoS_*x*_-Au photocatalysts is dependent on the Au amount (Supplementary Fig. 6). When the Au is precisely maintained at 3%, the resulting TiO_2_/MoS_*x*_-Au sample exhibits the highest H_2_O_2_-production rate with a value of 30.44 mmol g^−1^ h^−1^ (Fig. 3a and Supplementary Fig. 6), which is 25.4 and 1.3 times higher than that of TiO_2_ and TiO_2_/Au, respectively. However, increasing the Au content beyond this point to 5% leads to a reduction in H_2_O_2_ yield due to the light-shielding effect. According to the wavelength-dependent H_2_O_2_-evolution activity (Fig. 3b), the AQY of TiO_2_/MoS_*x*_-Au achieves an impressive value of 7.2% at 365 nm. The present H_2_O_2_-evolution performance is much higher than most reported results in TiO_2_-based photocatalysts or other inorganic photocatalysts (Supplementary Table S2). To investigate the application potential of present TiO_2_/MoS_*x*_-Au, its photocatalytic H_2_O_2_-evolution performance was further tested under different conditions. As exhibited in Fig. 3c and Supplementary Fig. 7, it is clear that there is almost no H_2_O_2_ generation over TiO_2_ in the air environment. In contrast, the H_2_O_2_ yield in the TiO_2_/MoS_*x*_-Au still maintains a high concentration (14.45 mmol g^−1^ h^−1^), indicating the great application potential of the TiO_2_/MoS_*x*_-Au photocatalyst. Besides, no significant decrease in the H_2_O_2_ concentration is observed after four cycles of photocatalytic reaction (Fig. 3d), revealing the robust reusability of the TiO_2_/MoS_*x*_-Au. From the above results, it can be concluded that the introduction of MoS_*x*_ mediator into TiO_2_/Au can effectively improve the photocatalytic H_2_O_2_-production activity.

### Photocatalytic mechanism of TiO_2_/MoS_*x*_-Au

**Fig. 4:**
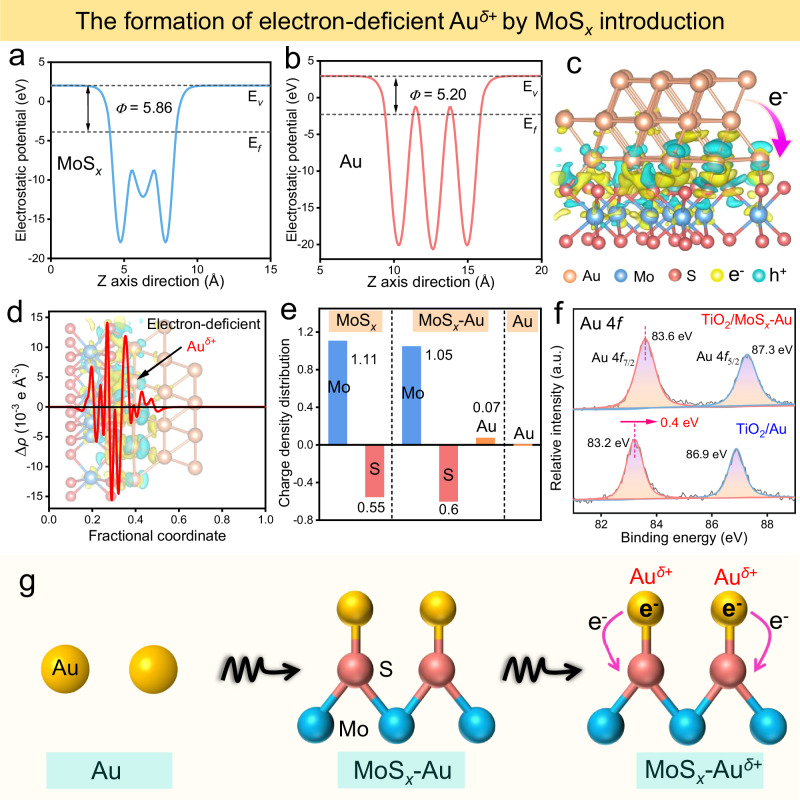
MoS_*x*_-induced electron-deficient Au^δ+^ formation and mechanism. **a**, **b** Calculated average potential profiles of MoS_*x*_ and Au. **c** The local charge density difference of MoS_*x*_-Au, where the light yellow and cyan areas represent electron accumulation and depletion, respectively. **d** Planar-averaged electron density difference ∆*ρ*(z) in MoS_*x*_-Au. **e** The charge density distributions of MoS_*x*_, MoS_*x*_-Au, and Au. **f** High-resolution XPS spectra of Au 4*f* in the TiO_2_/Au and TiO_2_/MoS_*x*_-Au. **g** Schematic illustration of the formation of electron-deficient Au^*δ*+^ sites in the MoS_*x*_-Au cocatalyst.

Considering the boosted H_2_O_2_-production activity, the effect of the MoS_*x*_ mediator on the Au electronic structure is primarily investigated by the first-principles calculations and XPS technology. For comparison, three slab models of Au, MoS_*x*_, and MoS_*x*_-Au are reasonably selected and optimized (Supplementary Fig. 8). Based on the optimized models, the work functions (*Φ*) of MoS_*x*_ (001) and Au (111) are calculated to be 5.86 and 5.20 eV, respectively (Fig. 4a, b). In this case, when Au is loaded onto the MoS_*x*_ surface, free electrons would migrate inevitably from Au nanoparticles to MoS_*x*_ (Supplementary Fig. 9)^45,46^, thus inducing the formation of electron-deficient Au^*δ*+^ sites. This electron transfer is further substantiated by examining the local charge density difference and the corresponding planar-averaged electron density difference (Fig. 4c, d)^47^. Obviously, the MoS_*x*_-Au cocatalyst shows a distinct electron-enriched region on the MoS_*x*_ side, while positive charges predominantly accumulate on Au atoms, leading to the production of an electron-deficient Au^*δ*+^ layer (Fig. 4d). To quantify the above charge transfer between Au and MoS_*x*_, Bader charge calculation was performed and shown in Fig. 4e. Clearly, they indicate a more negative charge density for S and Mo atoms in MoS_*x*_-Au compared to pure MoS_*x*_. Conversely, the charge density of Au atoms is slightly increased (+0.07) to produce Au^*δ*+^ active sites in the MoS_*x*_-Au cocatalyst. The formation of above electron-deficient Au^*δ*+^ can further be verified by XPS analysis (Fig. 4f). Compared with the TiO_2_/Au, a clear shift of binding energy (from 83.2 to 83.6 eV, ∆ = 0.4 eV) to a higher value is observed for Au 4*f* in the TiO_2_/MoS_*x*_-Au. In addition, the XPS peaks of S 2*p* (162.0 eV) and Mo 3*d* (231.6 eV) shift to lower values (∆= 0.6 eV for S 2*p*, ∆= 0.3 eV for Mo 3*d*) than those of TiO_2_/MoS_*x*_ (Supplementary Fig. 10), indicating the efficient electron transfer from Au to MoS_*x*_. The above electron transfer can also cause a slight change of binding energy for the Ti element (Supplementary Fig. 11). Unquestionably, both DFT calculation and experimental results strongly support that the introduction of MoS_*x*_ mediator can effectively modulate the Au electronic structure to induce the formation of electron-deficient Au^*δ*+^ sites in the MoS_*x*_-Au cocatalyst (Fig. 4g).

**Fig. 5:**
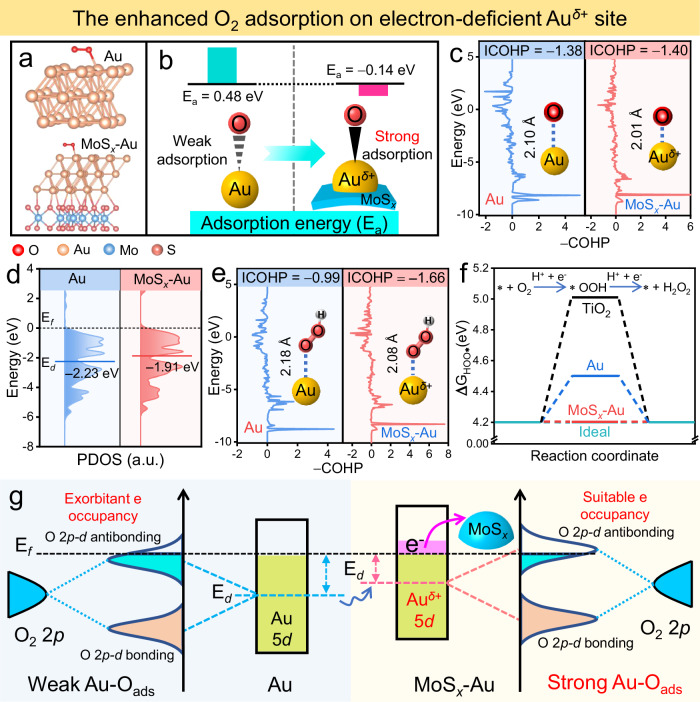
Modifying the electron structure of Au cocatalyst to decrease the antibonding-orbital occupancy of Au-O_ads_ for enhancing Au-O_ads_ bonds. **a** Optimized configurations for O_2_ adsorption on Au and MoS_*x*_-Au, and **b** the corresponding adsorption energies and schematic illustration about the improved O_2_-adsorption ability. **c** COHP analyses of O_2_ adsorption on Au and MoS_*x*_-Au. **d** PDOS diagrams of Au 5*d* orbitals in Au and MoS_*x*_-Au. **e** COHP analyses of *OOH adsorption on Au and MoS_*x*_-Au (* represents active site). **f** ∆*G*_HOO*_ over TiO_2_, Au and MoS_*x*_-Au. **g** Schematic diagram about the enhanced O_2_ adsorption on Au sites by decreasing antibonding-orbital occupancy via raising its *d*-band center.

The generation of electron-deficient Au^*δ*+^ impacts the O_2_ adsorption capability of TiO_2_/MoS_*x*_-Au photocatalyst, as evidenced by various analyses: O_2_ adsorption energy, crystal orbital Hamilton population (COHP), bonding distance analysis, and partial density of states (PDOS) calculations. Based on the optimized models in Fig. 5a and Supplementary Fig. 12, the O_2_ adsorption energies (*E*_a_) of Au sites before and after MoS_*x*_ introduction were first calculated (Fig. 5b). Clearly, the electron-deficient Au^*δ*+^ sites in the MoS_*x*_-Au cocatalyst exhibit more negative adsorption energy (*E*_a_ = −0.14 eV) than pure Au (*E*_a_ = 0.48 eV), indicating stronger O_2_ adsorption capability^48^. Further evidence of this enhanced adsorption is provided by COHP calculations^49^, revealing a smaller integrated COHP value (−1.40) for Au-O_ads_ bonds in MoS_*x*_-Au than in pure Au (−1.38), and shorter bond length (2.01 Å) compared to pure Au (2.10 Å), signifying stronger Au-O_ads_ bonding in electron-deficient Au^*δ*+^ sites (Fig. 5c)^50^. In this case, the *d*-band center mechanism helps explain this enhanced Au-O_ads_ bonding in the MoS_*x*_-Au^51^. As depicted in Fig. 5d, g, the *d*-band center of Au 5*d* in pure Au cocatalyst is −2.23 eV, significantly far away from E_f_. Consequently, the lower *d*-band center causes exorbitant antibonding-orbital occupancy, leading to a weak Au-O_ads_ bond. However, when Au is loaded on the MoS_*x*_ surface, the resulting *d*-band center of Au 5*d* in the MoS_*x*_-Au is closer to the *E*_f_ (−1.91 eV) than that of pure Au suggesting that modulating the electron structure of the Au cocatalyst to form electron-deficient Au^*δ*+^ sites can significantly elevate the *d*-band center (Fig. 5g). In this case, when O_2_ is adsorbed on the electron-deficient Au^*δ*+^ sites, the antibonding-orbital occupancy of Au-O_ads_ is decreased, resulting in a reinforced O_2_ adsorption. Therefore, the presence of MoS_*x*_ cocatalyst in the MoS_*x*_-Au can effectively raise the *d*-band center of Au^*δ*+^ sites for improved O_2_ adsorption, which is one of the essential steps for the following H_2_O_2_ production.

It is worth emphasizing that a suitable Au-O_ads_ bond can effectively contribute to the formation of Au-OOH_ads_ intermediate, thus greatly improving the selectivity and activity of photocatalytic H_2_O_2_ production (Supplementary Fig. 13). Hence, to explore the effect of improved Au-O_ads_ bonds on the formation of Au-OOH_ads_ intermediate in MoS_*x*_-Au cocatalyst, COHP calculation was carried out. As illustrated in Fig. 5e, both COHP value (−1.66) and bond length (2.08 Å, inset) of Au-OOH_ads_ in MoS_*x*_-Au are smaller than those in pure Au (−0.99 and 2.18 Å), forcefully manifesting that the MoS_*x*_-Au possess stronger Au-OOH_ads_ bonds, which is beneficial to improving the selectivity of photocatalytic H_2_O_2_ production. The above selectivity and activity of photocatalytic H_2_O_2_ production on MoS_*x*_-Au cocatalyst can further be confirmed by the free energy of *OOH intermediate (∆*G*_HOO*_) based on the optimized models in Supplementary Fig. 14^22^. As shown in Fig. 5f, the ∆G_HOO*_ values on TiO_2_ and Au sites are estimated to be 5.01 and 4.5 eV, respectively, which are significantly higher than the ideal ∆*G*_HOO*_ value (4.2 eV)^52^. These results suggest that TiO_2_ and Au have relatively weak adsorption and easier detachment for *OOH intermediates, leading to sluggish interfacial H_2_O_2_-production kinetics (Supplementary Fig. 13a). In contrast, the electron-deficient Au^*δ*+^ sites in the MoS_*x*_-Au cocatalyst showcase the best ∆G_HOO*_ values (4.2 eV), aligning with the ideal energy for *OOH adsorption and facilitating rapid H_2_O_2_ generation (Supplementary Fig. 13b).

**Fig. 6:**
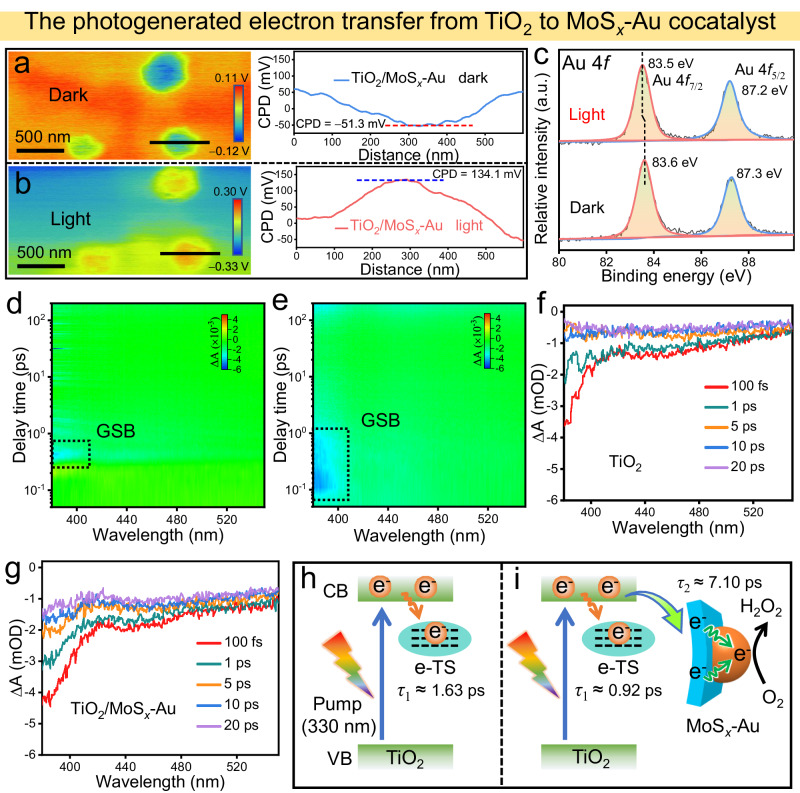
Photogenerated electron transfer mechanism and dynamics. **a**, **b** KPFM images and the corresponding surface potential profiles of the TiO_2_/MoS_*x*_-Au in the dark and light illumination, a 365 nm-LED light as the light source. **c** ISI-XPS spectra of Au 4*f* for TiO_2_/MoS_*x*_-Au before and after light illumination. Pseudocolor plots of **d** TiO_2_ and **e** TiO_2_/MoS_*x*_-Au (GSB represents ground-state bleaching). **f**, **g** Femtosecond transient absorption spectra of TiO_2_ and TiO_2_/MoS_*x*_-Au within 20 ps. All data were obtained under excitation of 330 nm and optical power of 600 μW cm^−2^. Schematic illustration of the decay pathways of photogenerated electrons in **h** TiO_2_ and **i** TiO_2_/MoS_*x*_-Au.

In addition to enhancing O_2_ adsorption for efficient H_2_O_2_ production, the MoS_*x*_-Au cocatalyst also promotes the rapid transfer of photogenerated electrons in the TiO_2_/MoS_*x*_-Au photocatalyst, which is supported by the subsequent Kelvin probe force microscopy (KPFM) and in situ XPS^53,54^. A scanning probe microscopy (SPM) system with KPFM was employed to analyze the distribution and transfer pathways of photogenerated electron-hole pairs in photocatalysts. The resulting AFM topography image, KPFM image, and the corresponding contact potential difference (CPD) profiles of TiO_2_ and TiO_2_/MoS_*x*_-Au are shown in Fig. 6a, b, and Supplementary Figs. 15 and 16. Obviously, the TiO_2_/MoS_*x*_-Au particles are observed, and a line scan across the above sample pre- and post-light irradiation is used to evaluate the CPD change. Upon light irradiation, the CPD value of TiO_2_ shows a slight increase from −14.7 to 8.6 mV owing to the spontaneous transfer of photogenerated holes onto the TiO_2_ surface (Supplementary Fig. 16)^55^. After the loading of MoS_*x*_-Au, the TiO_2_/MoS_*x*_-Au exhibits an obvious CPD value increase of about 185.4 mV (from −51.3 to 134.1 mV) during light irradiation, accompanied by a color change from blue to red owing to enhanced hole accumulation on the TiO_2_ surface (Fig. 6a, b), strongly indicating that the photogenerated electrons are efficiently transferred from TiO_2_ to MoS_*x*_-Au cocatalyst^56^. To further validate the above transfer of photogenerated electrons and their enrichment on the Au active sites of TiO_2_/MoS_*x*_-Au, in situ XPS was performed (Fig. 6c). The peaks of Au 4*f*_7/2_ and Au 4*f*_5/2_ in the TiO_2_/MoS_*x*_-Au are remarkably shift toward lower binding energies (from 83.6 eV to 83.5 eV, ∆ = 0.1 eV) upon light irradiation, suggesting that the photogenerated electrons are directionally transferred from TiO_2_ to MoS_*x*_-Au and mainly enriched on the electron-deficient Au^*δ*+^ sites, thereby promoting the photocatalytic H_2_O_2_-production kinetics^57^.

For a comprehensive understanding of electron-transfer dynamics in the TiO_2_/MoS_*x*_-Au, femtosecond transient absorption spectroscopy (fs-TAS) was carefully performed^58^. As shown in Fig. 6d, e and Supplementary Fig. 17a, a typical photobleaching peak (~380 nm) is displayed in the pseudocolor plots of TiO_2_, TiO_2_/Au, and TiO_2_/MoS_*x*_-Au. These signals are assigned to the ground-state bleaching (GSB), which reflects the excited state relaxation^59^. Further monitoring of the GSB signal (380 nm) within 20 ps reveals stronger intensity in the TiO_2_/Au (Supplementary Fig. 17b) and TiO_2_/MoS_*x*_-Au (Fig. 6g) compared to TiO_2_ (Fig. 6f), indicating enhanced electron enrichment in both TiO_2_/Au and TiO_2_/MoS_*x*_-Au^39^. Further analysis of the interfacial electron transfer involved fitting decay kinetics within 25 ps at 380 nm using biexponential equations, with fitting results of normalized curves shown in Supplementary Fig. 18 and Table S3. The short-lived *τ*_1_ corresponds to the electron trapping by electron trapping state (e-TS), while the long-lived *τ*_2_ is related to the interfacial electron transfer from TiO_2_ to cocatalyst. Meanwhile, *A*_1_ and *A*_2_ represent the decay proportion of photogenerated electrons during the electron trapping and transfer, respectively. Obviously, the TiO_2_ primarily undergoes a short-lived process within 25 ps under irradiation, and the corresponding *τ*_1_ is 1.63 ps, which is primarily attributed to electron trapping in the e-TS (Fig. 6h). Interestingly, the *τ*_1_ value in the TiO_2_/Au and TiO_2_/MoS_*x*_-Au significantly decreases to 0.36 and 0.92 ps, respectively, suggesting rapid transfer of a portion of photogenerated electrons from TiO_2_ to Au (*τ*_2_ = 5.88 ps) and MoS_*x*_-Au (*τ*_2_ = 7.10 ps) cocatalysts (Fig. 6i). Noticeably, the TiO_2_/MoS_*x*_-Au exhibits a larger *A*_2_ value (*A*_2_ = 39.6%) compared to the TiO_2_/Au (*A*_2_ = 32.3%), indicating more effective transfer of photogenerated electrons from TiO_2_ to Au facilitated by the MoS_*x*_ mediator^60^. The improved electron transfer on TiO_2_/MoS_*x*_-Au is well consistent with the results of photoelectrochemical and transient-state photoluminescence (TRPL) (Supplementary Fig. 19). These above results provide concertedly evidence that the MoS_*x*_-Au cocatalyst serves as an efficient platform for rapid transfer of photogenerated electrons to engage in the subsequent H_2_O_2_-production reaction at the electron-deficient Au^*δ*+^ sites (Fig. 6i), thus achieving high photocatalytic H_2_O_2_ yields.

Overall, a strategy of electronic structure modification for the Au cocatalyst has been proposed to effectively reinforce the Au-O_ads_ bonding at electron-deficient Au^*δ*+^ sites within the MoS_*x*_-Au cocatalyst, which can achieve an enhanced O_2_ adsorption for fast H_2_O_2_-production kinetics. As a result, an exceptional H_2_O_2_-production rate of 30.44 mmol g^−1^ h^−1^ has been achieved in the resulting TiO_2_/MoS_*x*_-Au, which is 25.4 and 1.3 times higher than that of TiO_2_ and TiO_2_/Au, respectively. Theoretical simulations and experimental results consistently support the notion that the introduction of MoS_*x*_ mediator induces the formation of electron-deficient Au^*δ*+^ active sites in the MoS_*x*_-Au cocatalyst by free-electron transfer from the Au cocatalyst to MoS_*x*_, which decreases the antibonding-orbital occupancy of the Au-O_ads_, thereby enhancing the O_2_-adsorption ability to realize efficient H_2_O_2_-production performance. In addition, the MoS_*x*_-Au cocatalyst can also provide an efficient platform for the rapid transfer and enrichment of photogenerated electrons from TiO_2_, leading to a distinct improvement of photocatalytic H_2_O_2_-production activity for the TiO_2_/MoS_*x*_-Au. This work emphasizes a feasible strategy for optimizing the O_2_-adsorption strength to efficiently accelerate H_2_O_2_-production kinetics, offering a very promising approach for the rational design of electronic structure for efficient artificial photosynthesis.

## Methods

### Preparation of TiO_2_/MoS_*x*_ photocatalyst

TiO_2_ photocatalyst (P25) was calcined at 550 °C for 2 h in a muffle furnace before being used. The TiO_2_/MoS_*x*_ sample was synthesized by one-step lactic acid-induced method, as schematically demonstrated in Supplementary Fig. 1. First, 624 μL (NH_4_)_2_MoS_4_ (0.02 mol/L) solution was dropped into 160 mL lactic acid solution (10 vol%) under stirring. In this case, the H^+^ was released from lactic acid and would induce the transformation of MoS_4_^2-^ into MoS_*x*_ colloidal nanoparticles. Subsequently, the as-prepared TiO_2_ nanoparticles (0.1 g) were dispersed into the above solution. After stirring for 2 h, a brown product was collected by centrifugation and washing with deionized water and ethanol. Finally, the obtained product was dried at 80 °C for 12 h. The resulting brown powder was denoted as TiO_2_/MoS_*x*_. In addition, the pure MoS_*x*_ product was also obtained by a similar synthesis route of the above TiO_2_/MoS_*x*_ in the absence of TiO_2_.

### Preparation of TiO_2_/MoS_*x*_-Au photocatalyst

The MoS_*x*_-Au modified TiO_2_ photocatalyst (TiO_2_/MoS_*x*_-Au) was synthesized by a two-step route, including the initial deposition of MoS_*x*_ on the TiO_2_ surface and the subsequently selective photodeposition of Au onto the MoS_*x*_ surface. First, 0.1 g of the MoS_*x*_/TiO_2_ was mixed with 80 mL ethanol aqueous solution (20 vol%) in a 100 mL three-necked flask. Then, a known amount of chloroauric acid (0.1 mol/L, HAuCl_4_·4H_2_O) was added. After the above system was evacuated with N_2_ for 20 min and then irradiated with a 300 W Xenon lamp for 1 h, the resultant suspension was collected by centrifugated, rinsed, and dried at 80 °C for 12 h to obtain the final TiO_2_/MoS_*x*_-Au. To investigate the effect of Au amount on the structure and photocatalytic performance, the amount of Au in the TiO_2_/MoS_*x*_ was controlled to be 1, 1.5, 2, 3, and 5 wt%, respectively, and the resultant sample was referred to be TiO_2_/MoS_*x*_-Au-*X*% (*X* is the amount of Au).

### Preparation of TiO_2_/Au photocatalyst

Au nanoparticle-loaded TiO_2_ (TiO_2_/Au) was prepared by a photodeposition method. First, 0.1 g of the TiO_2_ was dispersed into a 100 mL of ethanol aqueous solution (20 vol%). Subsequently, a known amount of chloroauric acid (0.1 mol/L, HAuCl_4_·4H_2_O) was added dropwise to the above mixture solution. Before irradiation, the above system was bubbled with N_2_ for 20 min and then irradiated for 1 h. Finally, a light-purple product was collected by centrifugated, rinsed, and dried at 60 °C for 12 h. The resulting powder was denoted as TiO_2_/Au.

### Characterization

The microstructure of the samples was characterized by transmission electron microscopy (TEM; Thermal Fisher Talos F200X). X-ray photoelectron spectroscopy (XPS) was performed on a Thermo Scientific ESCALA 210 XPS spectrometer system (USA) with 300 W Al Kα radiation to survey the elemental composition and valence states of these photocatalysts. The X-ray diffraction (XRD) patterns of the samples were obtained on an X-ray diffractometer (Shimadzu XRD-6100) with Cu Kα radiation. The elemental content was performed by inductively coupled plasma optical emission spectrometry (ICP-OES). The ultraviolet-visible spectra (UV-vis DRS) were obtained on a UV-vis spectrophotometer (UV-2600i, Shimadzu, Japan). Time-resolved photoluminescence (TRPL) spectra were acquired on a fluorescence lifetime spectrophotometer (FLS 1000, Edinburgh, UK). The photo-irradiated Kelvin probe force microscopy (KPFM) (SPM-9700, Shimadzu, Japan) was carried out to test the contact potential difference of the samples.

### Photocatalytic H_2_O_2_ production test

Photocatalytic H_2_O_2_-production activity was examined in an O_2_-saturated aqueous solution with ethanol as a hole scavenger, and a 300 W Xenon arc lamp was selected as the light source. First, 10 mg of as-prepared photocatalyst and 100 mL of ethanol solution (10 vol%) were mixed in a 100 mL three-necked flask reactor. Before irradiation, the system was purged with oxygen for 30 min to obtain an O_2_-saturated solution. During the photocatalytic H_2_O_2_-production test, 1 mL of solution was sampled from the reactor. Finally, the H_2_O_2_ concentration was examined via an iodometry method by using a UV-visible spectrophotometer (UV-1240, Japan). The concentration of H_2_O_2_ was calculated by the equation ($y{{\mbox{=}}}0.00771x{{\mbox{+}}}0.0218$), and the reaction mechanism was shown in Eq. (1). The absorbance of I_3_^-^ at 350 nm can be recorded by UV-vis spectroscopy.

1 $${{{\mbox{H}}}}2{{{\mbox{O}}}}_{2}+3{{{{{{\rm{I}}}}}}}^{-}+2{{{\mbox{H}}}}^{+}\to {{{{{{\rm{I}}}}}}}_{3}^{-}+2{{{\mbox{H}}}}_{2}{{\mbox{O}}}$$

### Photoelectrochemical measurements

The photoelectrochemical (PEC) properties were assessed using an electrochemical workstation (CHI 760E, China) within a three-electrode system. The working electrode was prepared by applying the photocatalyst onto a 1.0 cm^2^ FTO glass substrate. The Ag/AgCl (in a saturated KCl solution) and Pt foil were designated as the reference and counter electrodes, respectively. The PEC assessments were carried out in a 0.5 M Na_2_SO_4_ solution using a 300 W Xenon arc lamp for illumination.

### Average decay time (*τ*_average_) calculation

The decay curves of as-prepared samples from the TRPL can be effectively fitted using the following biexponential Eq. (2), and the fluorescent lifetime (${\tau }_{{{\mbox{a}}}}$) is calculated by Eq. (3).

2 $${A}_{(t)}={A}_{(0)}+{A}_{1}\exp (-t/{\tau }_{1})+{A}_{2}\exp (-t/{\tau }_{2})$$

3 $${\tau }_{{{{{{\rm{a}}}}}}}=({A}_{1}{{\tau }_{1}}^{2}+{A}_{2}{{\tau }_{2}}^{2})/({A}_{1}{\tau }_{1}+{A}_{2}{\tau }_{2})$$

where ${A}_{1}$ and ${A}_{2}$ represent the weight factors, while ${\tau }_{1}$ and ${\tau }_{2}$ are the short and long fluorescent lifetimes, respectively.

### Apparent quantum yield (AQY) calculation

The AQY measurement was conducted in an O_2_-saturated aqueous solution with ethanol as a hole scavenger by utilizing a 300 W Xenon arc lamp as the light source. In detail, the as-prepared photocatalyst (10 mg) and ethanol solution (100 mL, 10 vol%) were mixed in a 100 mL three-necked flask reactor, which was oxygenated for 30 min to obtain the O_2_-saturated solution.

The apparent quantum yields for H_2_O_2_ were calculated from the following Eq. (4):

4 $$\eta=\frac{{N}_{e}}{{N}_{p}}\times 100\%=\frac{2\times M\times {N}_{{{{{{\rm{A}}}}}}}\times h\times c}{S\times P\times t\times \lambda }\times 100\%$$

where *M* represents the amount of produced H_2_O_2_ molecules (mol), *N*_A_ is the Avogadro constant (6.022 × 10^23^/mol), *h* is the Planck constant (6.626 × 10 ^−34^ J s), *c* is the speed of light (3 × 10^8 ^m/s), *S* is the irradiation area (15.83 cm^2^), *P* is the average intensity of irradiation (365 nm, 13.35 mW/cm^2^), *t* is the irradiation time (s), and *λ* is the wavelength of the incident monochromatic light (nm).

### Ultrafast transient absorption (TA) tests

Femtosecond transient absorption spectra of the as-prepared photocatalysts were obtained on a pump-probe system (Helios, Ultrafast System) with a maximum time delay of ~8 ns using a motorized optical delay line under ambient conditions. The 330 nm-pump pulses (600 μW average at tested samples) were generated by the 1 kHz regenerative amplifier (Coherent Libra, 800 nm, 35 fs, 5 mJ) in an optical parametric amplifier (OPerA Solo), seeded with a mode-locked Ti: sapphire oscillator (Coherent Vitara, 800 nm, 80 MHz) and pumped with an LBO laser (Coherent Evolution-50C, 1 kHz system). To generate the white light from 320 to 750 nm, the 800 nm-femtosecond pluses were pumped by a constantly rotating sapphire crystal.

### Computational details

The density functional theory (DFT) calculations were performed using the Vienna Ab initio Simulation Package (VASP) with the generalized gradient approximation (GGA) employing the revised Perdew–Burke–Ernzerhof (PBE) functional for the exchange-correlation interaction. The convergence threshold for total energy converged within 10^−4^ eV/atom, and the average force was 0.01 eV/Å. Grid integration utilized a cutoff energy of 450 eV, and projector-augmented wave (PAW) potentials characterized the ion cores. To rationalize the calculation, unsaturated sulfur (S) atoms were obtained on the edge of MoS_*x*_ model by creating a vacuum layer in the *y*-axis direction, which displayed a similar local coordination structure as amorphous MoS_*x*_ cocatalyst. Moreover, (3 × 3 × 1) Monkhorst-Pack grids and (2 × 2) surface cells were used for oxygen adsorption. The adsorption energy *E*_ads_ was defined as ${E}_{{{{{{\rm{ads}}}}}}}={E}_{{{{{{\rm{total}}}}}}}-{E}_{{{{{{\rm{surface}}}}}}}-{E}_{{{{{{\rm{O}}}}}}2}$, where *E*_total_, *E*_surface_, and *E*_O2_ represent the energy of adsorption configurations, the energy of metallic surfaces, and the energy of molecular O_2_, respectively. In addition, the ΔG of HOO∗ intermediate on the surface was calculated by the equation $G=E+{ZPE}-{TS}$, where *E* is the total energy, *ZPE* is the zero-point energy, *T* is the temperature (298.15 K), and *S* is the entropy. Several configurations of the adsorbed models were considered in the simulation, and the most favorable ones are presented based on the adsorption energy.

### Supplementary information

Supplementary Information

Peer Review File

### Source data

Source data

## Data Availability

Data is available from the authors on request. All data generated in this study are provided in the Source data file. Source data are provided with this paper.
