# Supplementary material for: Enhancing photocatalytic H2O2 production with Au co-catalysts through electronic structure modification
Source: Nat Commun. 2024 Apr 13;15:3212. doi: 10.1038/s41467-024-47624-7 (PMC11016070; doi:10.1038/s41467-024-47624-7)
Supplement: Supplementary file 1 — Supplementary Information [file 41467_2024_47624_MOESM1_ESM.pdf]

Supplementary Information

Enhancing photocatalytic H_2O_2 production with Au co-catalysts through electronic structure modification

*Xidong Zhang[1], Duoduo Gao[2], Bicheng Zhu[1], Bei Cheng[3], Jiaguo Yu[1], Huogen Yu[1] **

[1] Laboratory of Solar Fuel, Faculty of Materials Science and Chemistry, China University of Geosciences, 68 Jincheng Street, Wuhan, 430078, P.R. China

[2] State Key Laboratory of Silicate Materials for Architectures, Wuhan University of Technology, 122 Luoshi Road, Wuhan 430070, P. R. China

[3] State Key Laboratory of Advanced Technology for Material Synthesis and Processing, Wuhan University of Technology, Wuhan 430070, PR China

*Correspondence: yuhuogen@cug.edu.cn

Supplementary Table S1. Compositions (wt%) of the as-prepared samples based on the ICP-OES results.

Samples	elements	Percentage (wt%)
TiO_2/Au	Au	3.10
	Au	3.34
TiO_2/MoS_x-Au	Mo	0.62
	S	0.80

Supplementary Table S2. Comparison of various photocatalytic materials and their corresponding H_2O_2-production rates.

Catalysts	Reactant solution	Light source	Rate of H_2O_2 production (mmol g^{-1} h^{-1})	Ref.
PEI/C_3N_4	H_2O	AM 1.5	0.2081	(1)[1]
PCN-NaCA-2	3.5% glycerol	AM 1.5	18.7	(2)[2]
$Ni_{4\%}/O_{0.2}$tCN	10% ethanol	300 W Xe lamp λ>420 nm	2.464	(3)[3]
AKMT	10% ethanol	300 W Xe lamp λ>420 nm	2.733	(4)[4]
5Cv@g-C_3N_4	10% ethanol	300 W Xe lamp λ>420 nm	7. 01	(5)[5]
CoPc-BTM-COF	10% ethanol	300 W Xe lamp λ>400 nm	2.096	(6)[6]
TC/g-CN/BOC	5% IPA	300 W Xe lamp	1.275	(7)[7]
ZnO/WO_3	10% ethanol	300 W Xe lamp	6.788	(8)[8]
TiO_2@NSG	10% ethanol	300 W Xe lamp	1.746	(9)[9]
$Zn_3In_2S_6$	Acetonitrile 25 mm THIQs	300 W Xe lamp λ>400 nm	66.4	(10)[10]
$Au_{0.1}Ag_{0.4}/TiO_2$	4% ethanol	450 W Hg lamp λ>280 nm	3.4	(11)[11]
Cu@Au/$BiVO_4$	5% methanol	12.5 mW LED lamp λ=420 nm	0.118	(12)[12]
C_3N_4-Au/$BiVO_4$	0.2 M citrate buffer solution	50 mW LED lamp λ=420 nm	0.676	(13)[13]
$BiVO_4$/AuPd	0.2 M citrate	20 mW LED	1.145	(14)[14]

	buffer solution	lamp λ=420 nm		
C_3N_4/Au	10% ethanol	300 W Xe lamp λ>420 nm	0.017	(15)[15]
WO_3/Au	4% methanol	400 W metal halide lamp λ>420 nm	0.111	(16)[16]
OVs-BiOBr-Au	5% formic acid	300 W Xe lamp λ>420 nm	0.127	(17)[17]
TiO_2/MoS_x-Au	10% ethanol	300 W Xe lamp	30.44	This work

Supplementary Table S3. Fitted parameters of the fs-TAS decay curves of TiO_2, TiO_2/Au, and TiO_2/MoS_x-Au.

Samples	τ_1 (ps)	A_1 (fraction)	τ_2 (ps)	A_2 (fraction)
TiO_2	1.63	-	-	-
TiO_2/Au	0.36	67.7	5.88	32.3
TiO_2/MoS_x-Au	0.92	60.4	7.10	39.6

Supplementary Figures

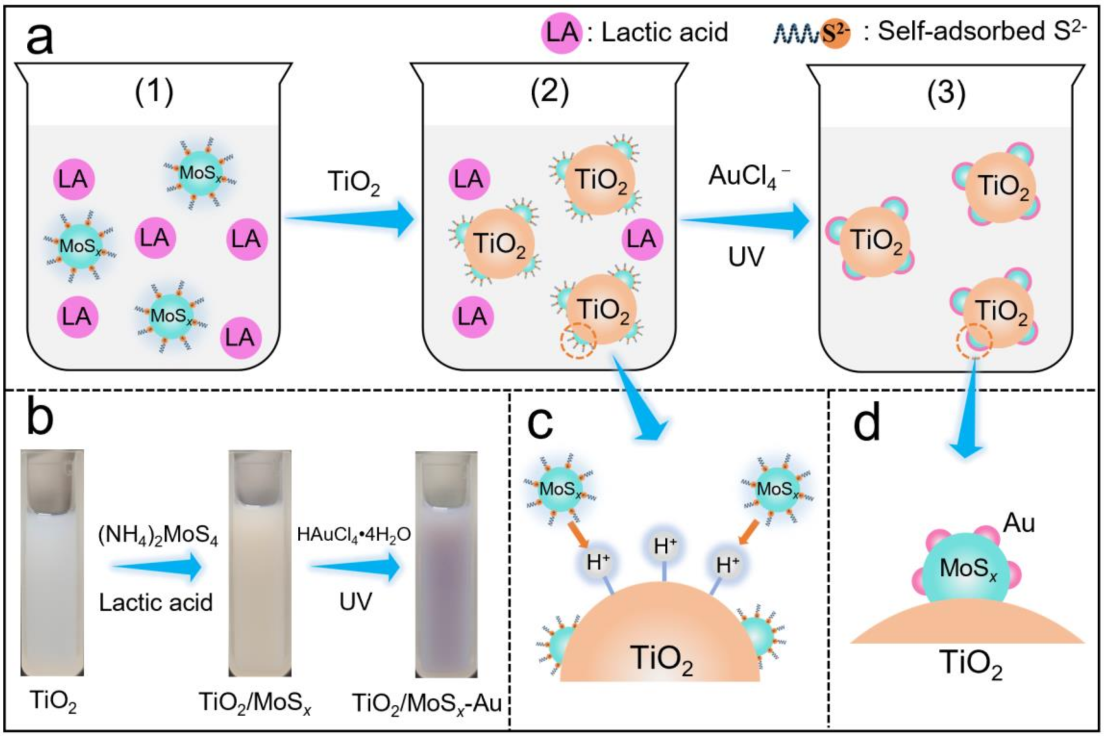

Supplementary Figure 1. The synthesis of TiO_2/MoS_x-Au photocatalyst. a Schematic diagram illustrating the synthesis progress of TiO_2/MoS_x-Au photocatalysts and (**b**) their corresponding sample photographs. The structure diagrams of (**c**) TiO_2/MoS_x and (**d**) TiO_2/MoS_x-Au.

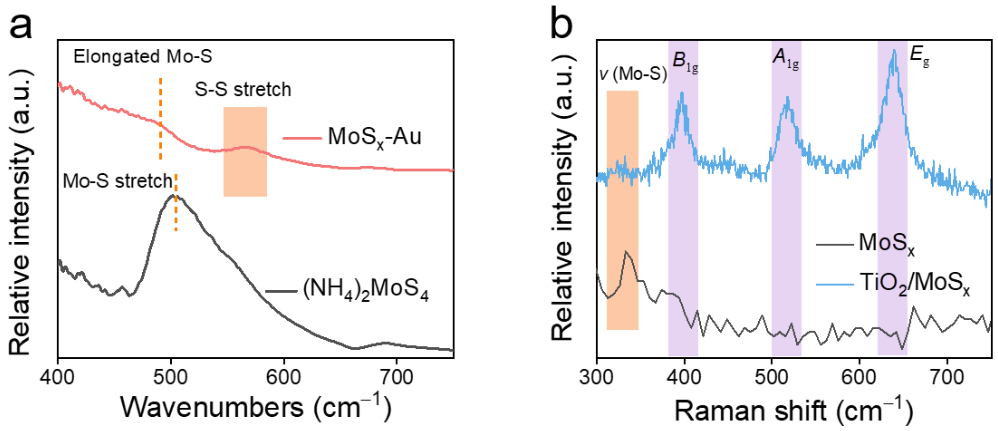

Supplementary Figure 2. FTIR and Raman spectra results. a FTIR spectra of $(NH_4)_2MoS_4$ and MoS_x-Au. **b** Raman spectra of MoS_x and TiO_2/MoS_x.

To validate the efficient synthesis of MoS_x, FTIR spectra of $(NH_4)_2MoS_4$ and MoS_x-Au were acquired and displayed in Supplementary Fig. 2a. A significant FTIR peak at ca. 500 cm^{-1} was presented in the $(NH_4)_2MoS_4$, which is attributed to the Mo-S vibration. Notably, a new S-S vibration peak was existed in the MoS_x-Au, indicating the successful hydrolysis of $(NH_4)_2MoS_4$ to produce MoS_x in the lactic acid solution. In addition, the Raman spectra of MoS_x and TiO_2/MoS_x were further obtained to confirm the efficient synthesis of MoS_x (Supplementary Fig. 2b). Obviously, a significant Raman peak at ca. 330 cm^{-1} was observed in MoS_x, which is ascribed to the Mo-S stretching vibration. However, other characteristic Raman peaks of out-of-plane (A_{1g}) and in-plane (E_{2g}^{1}) phonon modes for crystal MoS_2 were absent, revealing the amorphous structure of present MoS_x. Noticeably, a Mo-S stretching vibration was also found in the resulting TiO_2/MoS_x, indicating that MoS_x has been successfully deposited on the TiO_2 surface.

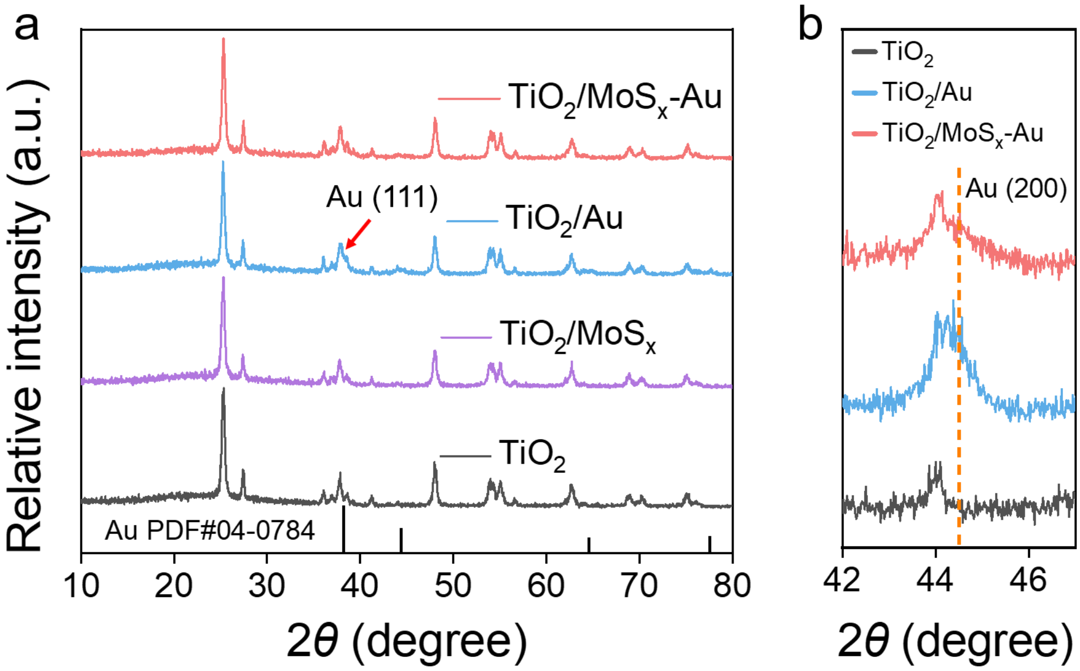

Supplementary Figure 3. Phase structure of the samples. a, b XRD patterns of as-prepared samples and their corresponding Au diffraction peak.

The XRD pattern of pure TiO_2 presented a typical mixing phases of anatase and rutile. In addition to the TiO_2 phase, both the TiO_2/Au and TiO_2/MoS_x-Au samples exhibited distinct diffraction peaks at ca. 38.1° and 44.3°, which can be attributed to the metallic Au. Moreover, no diffraction peask about MoS_x could be found in both of the TiO_2/MoS_x and TiO_2/MoS_x-Au photocatalysts, suggests the amorphous structure of MoS_x.

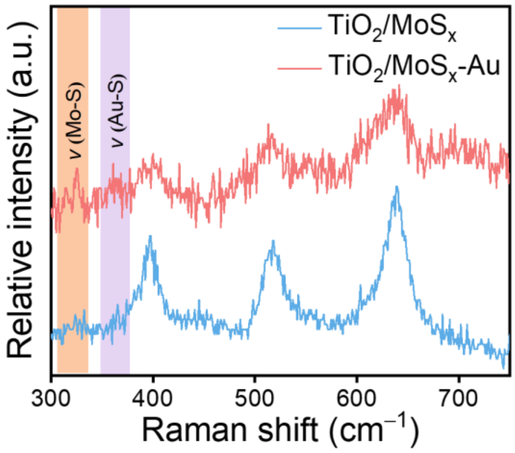

Supplementary Figure 4. Group vibration and rotation analysis. Raman spectra of TiO_2/MoS_x and TiO_2/MoS_x-Au.

To verify the S-induced selective deposition of Au nanoparticles on the MoS_x surface, Raman spectra of the TiO_2/MoS_x and TiO_2/MoS_x-Au were exhibited in Supplementary Fig. 4. As expected, both the TiO_2/MoS_x and TiO_2/MoS_x-Au showed a typical Mo-S stretching vibration. Noteworthily, an obvious characterization peak of Au-S bonds at 360.5 cm^{-1} was found in the TiO_2/MoS_x-Au, suggesting that the Au nanoparticles were successfully deposited onto the MoS_x surface.

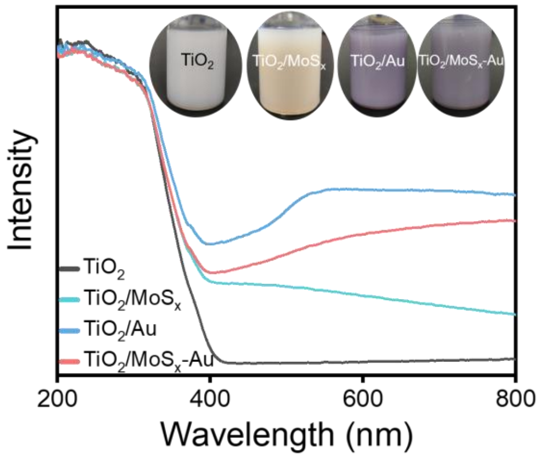

Supplementary Figure 5. The optical absorption performance. UV-vis absorption spectra of various samples and their corresponding photographs.

A typical surface plasmon resonance (SPR) absorption of Au (ca. 540 nm) was presented in the TiO_2/Au and TiO_2/MoS_x-Au photocatalysts, suggesting that Au nanoparticles were successfully deposited on the surfaces of both TiO_2 and TiO_2/MoS_x.

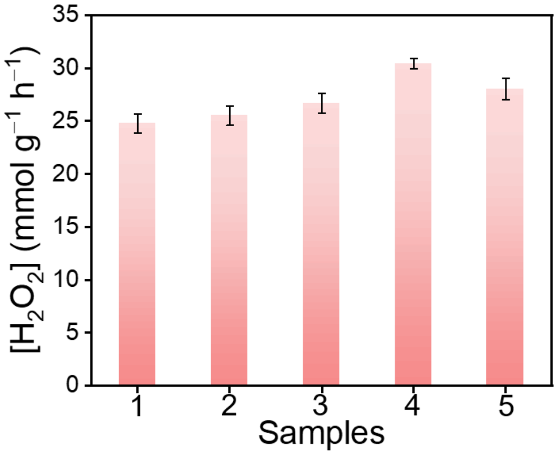

Supplementary Figure 6. Photocatalytic H_2O_2-production performance of TiO_2/MoS$_x$-Au with different Au content. (1) TiO_2/MoS$_x$-Au-1%, (2) TiO_2/MoS$_x$-Au-1.5%, (3) TiO_2/MoS$_x$-Au-2%, (4) TiO_2/MoS$_x$-Au-3%, (5) TiO_2/MoS$_x$-Au-5%. The error bars (mean ± standard deviation) were calculated based on three independent photocatalytic experiments.

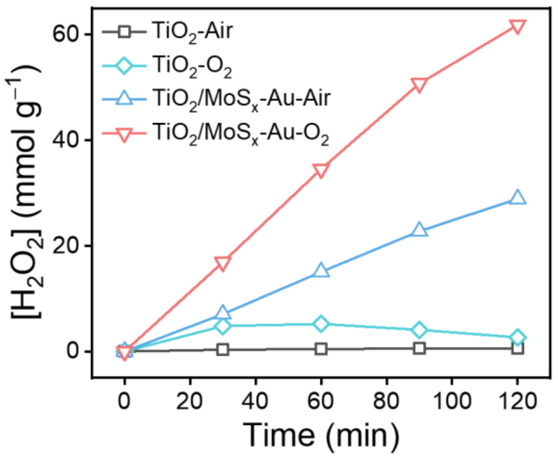

Supplementary Figure 7. **Photocatalytic H_2O_2-production activities in different conditions.** Time-dependent H_2O_2-evolution activity of TiO_2 and TiO_2/MoS_x-Au in the O_2-saturated and atmospheric conditons.

Under atmospheric conditions, there was almost no H_2O_2 generation over TiO_2, while the TiO_2/MoS_x-Au still exhibited a higher photocatalytic performance.

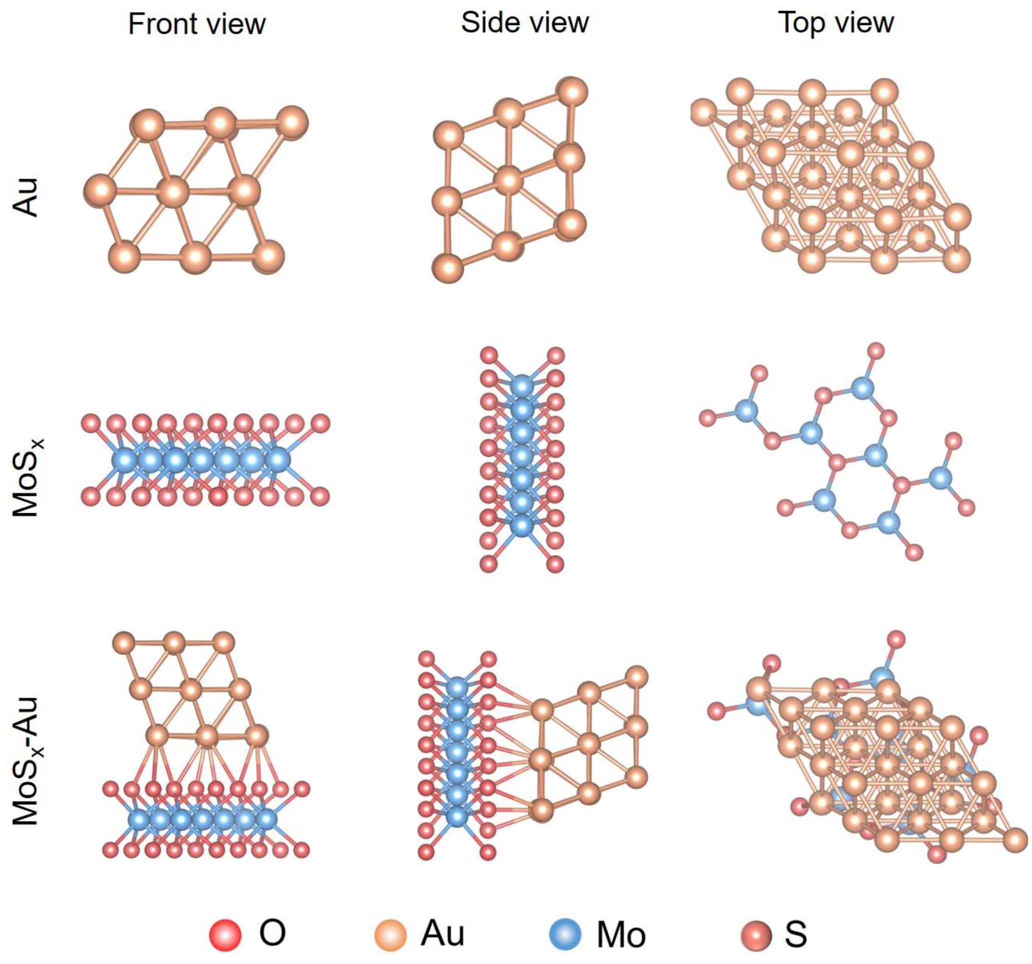

Supplementary Figure 8. Atomic structure models of various samples. Structure models of Au, MoS_x, and MoS_x-Au cocatalysts after optimization.

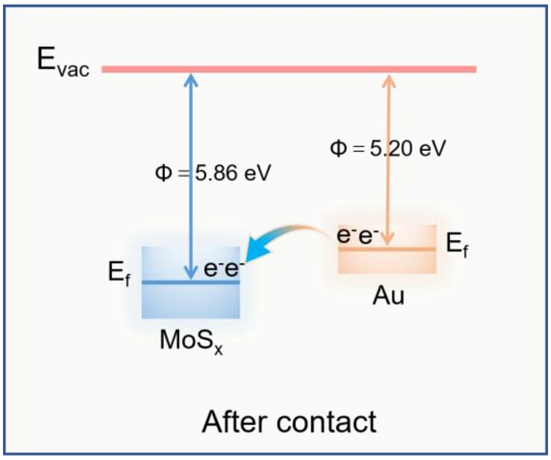

Supplementary Figure 9. Electron transfer between MoS$_x$ and Au. Schematic diagram illustrating the electron transfer between MoS$_x$ and Au due to their different work function.

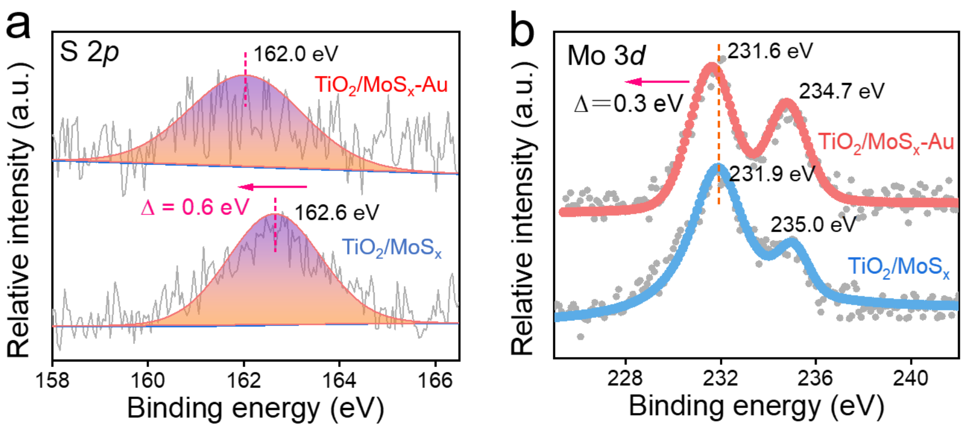

Supplementary Figure 10. Free electron transfer between MoS_x and Au. High-resolution XPS spectra of (**a**) S 2*p* and (**b**) Mo 3*d* for TiO_2/MoS_x and TiO_2/MoS_x-Au samples.

Compared with the TiO_2/MoS_x, the XPS peaks of both S 2*p* and Mo 3*d* in the TiO_2/MoS_x-Au obviously shifted to lower binding energies (Δ= 0.6 eV for S 2*p*, Δ= 0.3 eV for Mo 3*d*), suggesting the effective transfer of free electrons from Au to MoS_x.

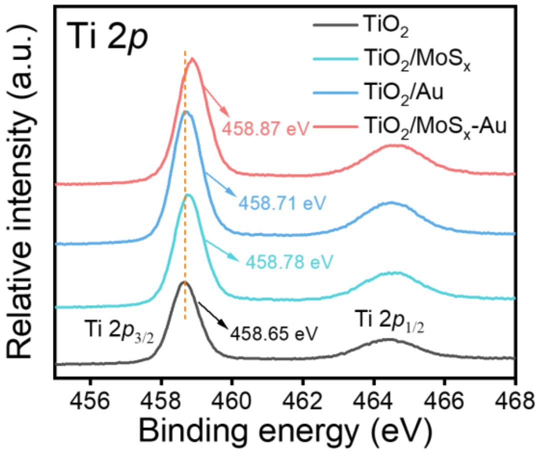

Supplementary Figure 11. Free-electron transfer between TiO_2 and various cocatalysts. High-resolution XPS spectra of Ti 2*p* in various photocatalysts.

Compared with bare TiO_2, the binding energy of Ti 2*p* in both TiO_2/MoS_x and TiO_2/Au presented a slight shift, while that of the TiO_2/MoS_x-Au clearly showed a distinct shift (Δ = 0.22 eV) to a higher value.

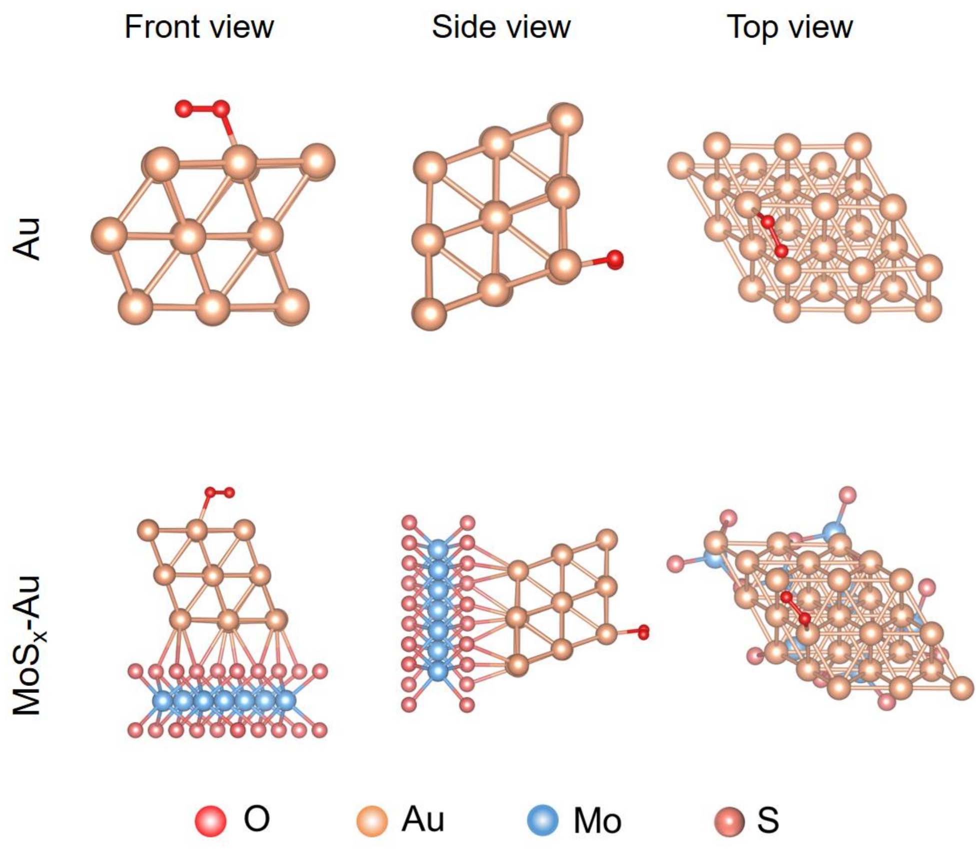

Supplementary Figure 12. The O_2-adsorption models of various samples. The optimized O_2-adsorption structures on the Au and MoS_x-Au cocatalysts (front, side, and top view).

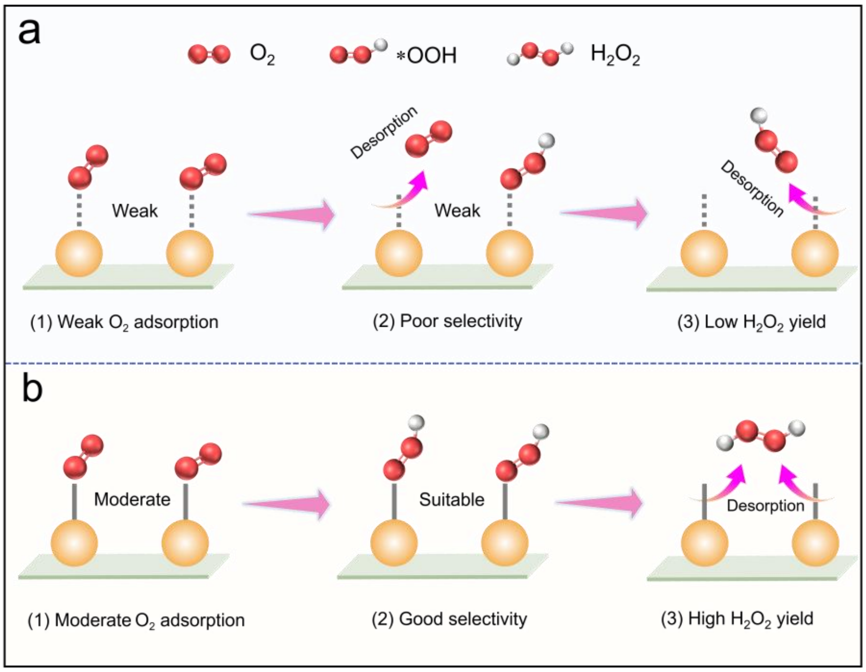

Supplementary Figure 13. Photocatalytic oxygen-reduction reaction for H_2O_2 production. **a** weak O_2 adsorption (low H_2O_2 yield) and (**b**) moderate O_2 adsorption (high H_2O_2 yield), * represents active site.

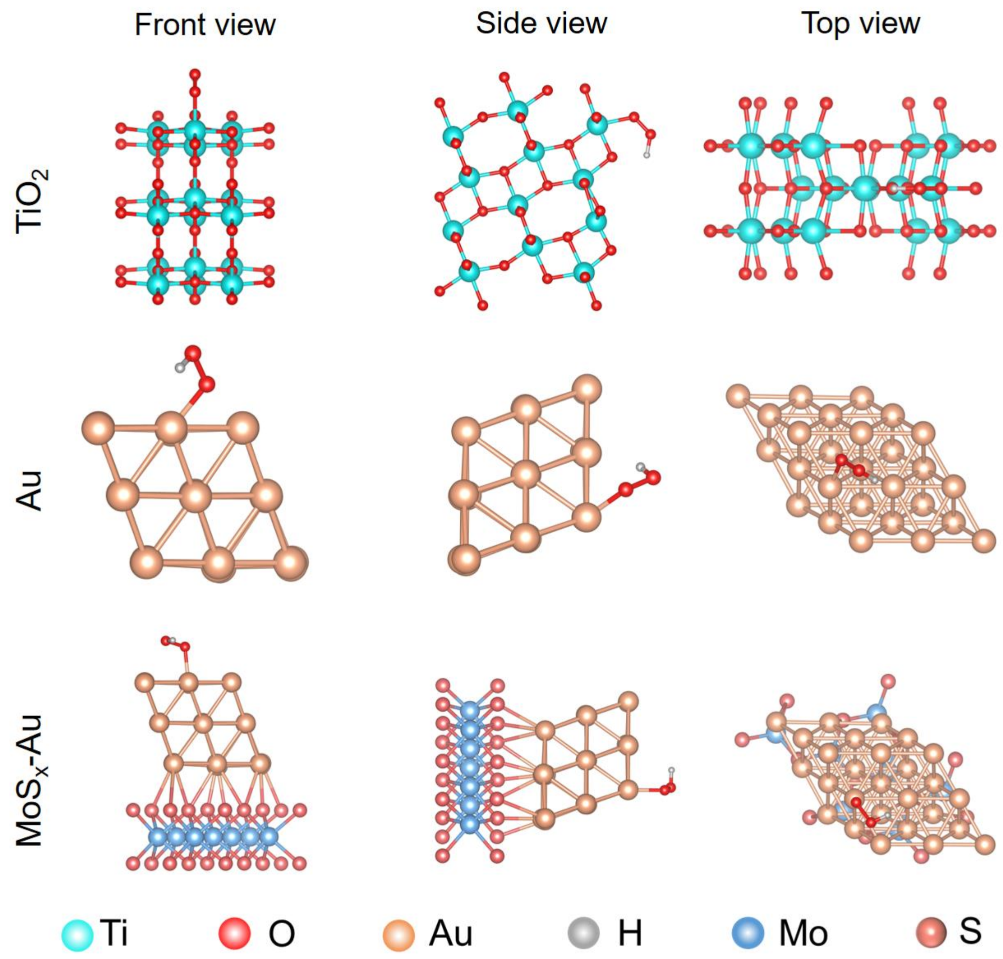

Supplementary Figure 14. The OOH-adsorption models of various samples. The optimized *OOH-adsorption structures on TiO_2, Au, and MoS_x-Au (front, side, and top view).

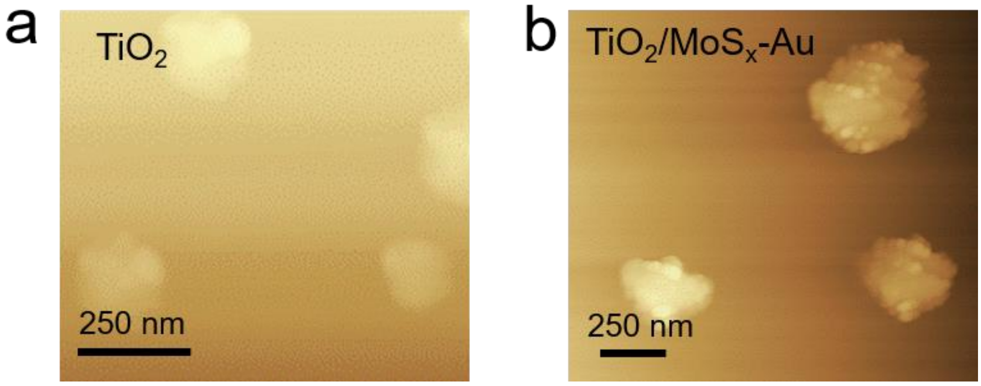

Supplementary Figure 15. The morphology of various samples. AFM topographies of (**a**) TiO_2 and (**b**) TiO_2/MoS_x-Au.

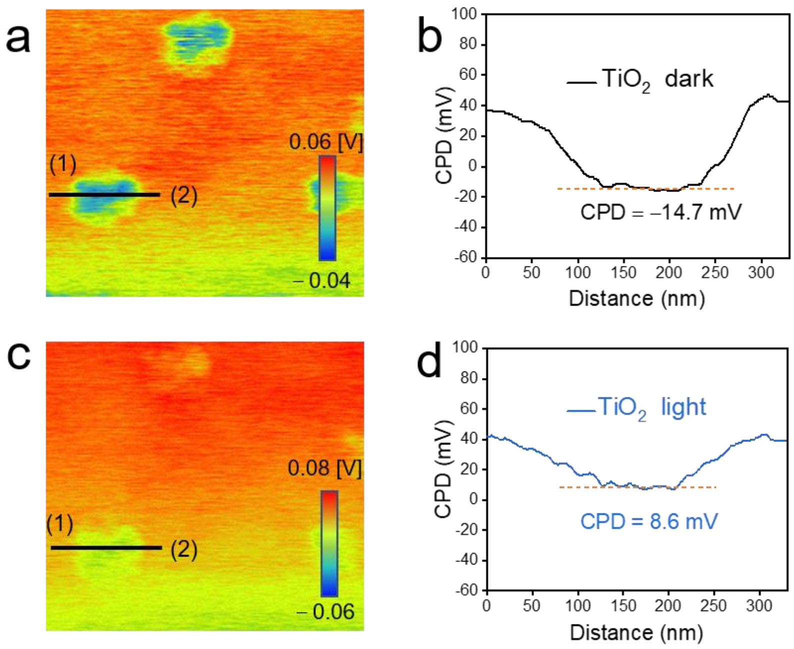

Supplementary Figure 16. The surface potential difference of TiO_2 under different conditions. KPFM images and the corresponding contact potential difference profiles of the TiO_2 in the (**a, b**) dark and (**c, d**) light illumination, a 365 nm-LED light as the light source.

The CPD value of TiO_2 was acquired synchronously in both dark and light conditions, and the CPD represented the contact potential difference between the sample and tip. The KPFM images and CPD profiles of TiO_2 were displayed in Supplementary Fig. 16. The position of (1) and (2) corresponded to highly oriented pyrolytic graphite (HOPG), while the region between them was TiO_2 (Supplementary Figs. 16a and c). Under 365 nm light irradiation, the CPD value of TiO_2 showed a slight increase of 23.3 mV (from −14.7 mV to 8.6 mV), and the color changed from blue to light yellow. The increased positive signal can be ascribed to the spontaneous migration of photogenerated holes onto the TiO_2 surface in the presence of light illumination.

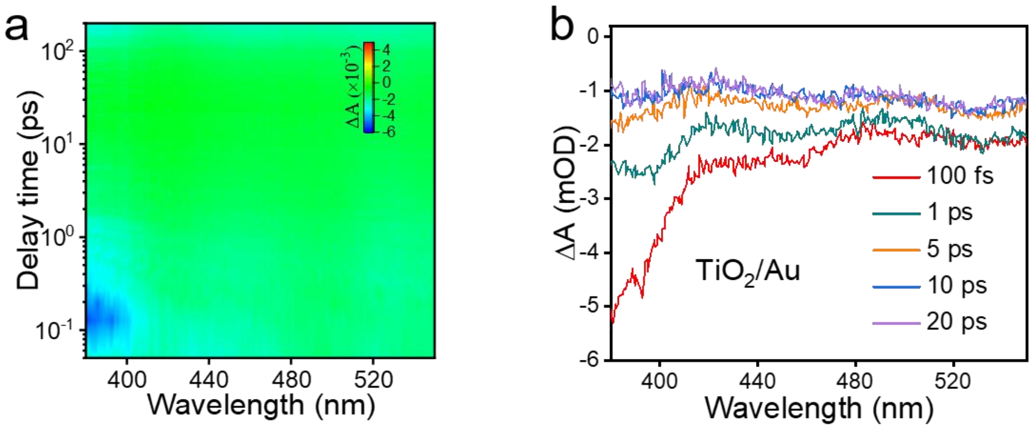

Supplementary Figure 17. Femtosecond transient absorption spectra results. a Pseudocolor plot of TiO_2/Au. **b** Femtosecond transient absorption spectra of TiO_2/Au within 20 ps. The change in absorbance (ΔA) is defined as the optical density (OD) difference, and the data was obtained under excitation of 330 nm and optical power of 600 μW cm^{-2}.

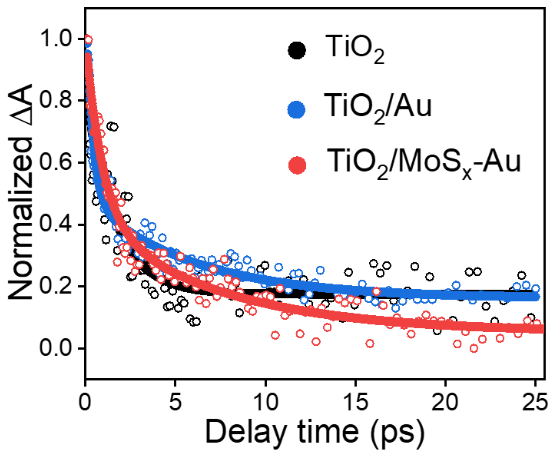

Supplementary Figure 18. Decay dynamics of photogenerated charges. Normalized decay kinetic curves of TiO_2, TiO_2/Au, and TiO_2/MoS_x-Au at 380 nm for the electron quenching processes.

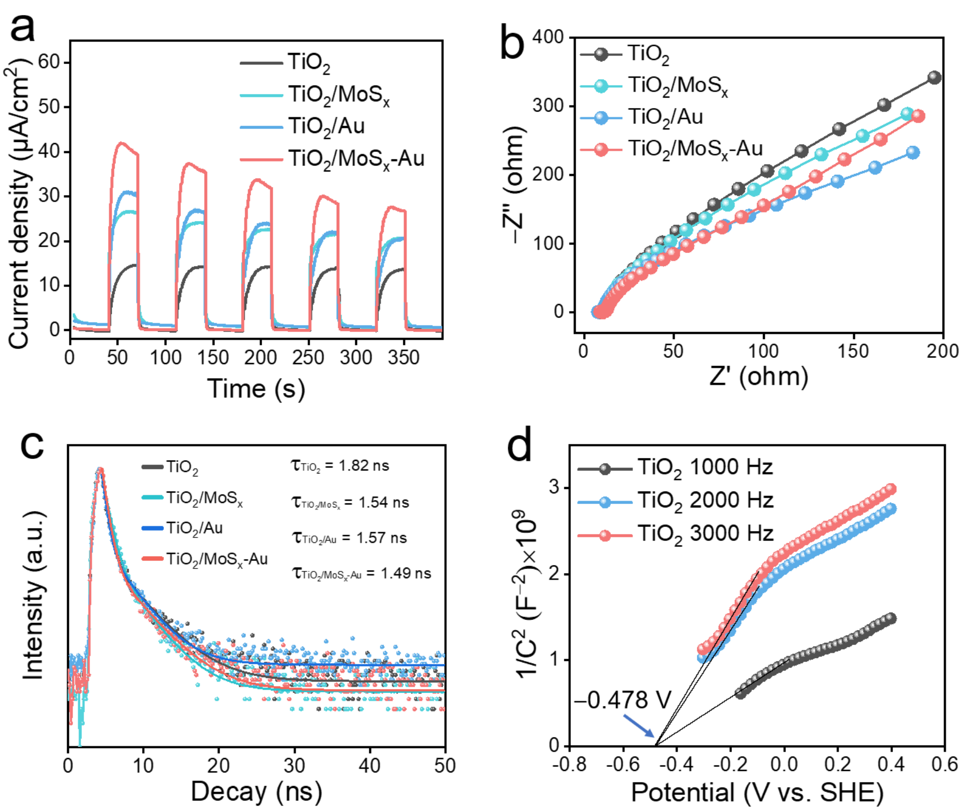

Supplementary Figure 19. Photoelectrochemical results and transient-state photoluminescence (TRPL) spectra. a Transient photocurrent response curves, **b** Nyquist plots, **c** TRPL spectra of TiO_2, TiO_2/MoS_x, TiO_2/Au, and TiO_2/MoS_x-Au, **d** Mott-Schottky plots of TiO_2 in 0.5 M Na_2SO_4 solution.

Transient photocurrents and electrochemical impedance spectra (EIS) were acquired to evaluate the photoelectrochemical properties of the resulting samples. As exhibited in Supplementary Figs. 19a and b, the TiO_2/MoS_x, TiO_2/Au, and TiO_2/MoS_x-Au displayed higher photocurrent densities and smaller EIS compared to pure TiO_2. Notably, the TiO_2/MoS_x-Au showed the highest photocurrent density, suggesting that the MoS_x-Au cocatalyst on the TiO_2 surface can promote the charge-carrier separation of TiO_2 more effectively. TRPL analysis was further conducted to investigate the

separation, migration, and lifetime of photogenerated charge carriers. TiO_2 decorated with cocatalysts showed smaller average PL lifetimes compared to TiO_2 (1.82 ns). Noteworthily, the TiO_2/MoS_x-Au photocatalyst exhibited the shortest average lifetime (1.49 ns) (Supplementary Fig. 19c), indicating an efficient charge transfer from TiO_2 to MoS_x-Au cocatalyst. In addition, according to the Mott-Schottky (M-S) results (Supplementary Fig. 19d), TiO_2 was thermodynamically capable of converting O_2 to H_2O_2.

Supplementary References

1. Zeng, X. et al. Simultaneously tuning charge separation and oxygen reduction pathway on graphitic carbon nitride by polyethylenimine for boosted photocatalytic hydrogen peroxide production. *ACS Catal.* **10**, 3697-3706 (2020).
2. Zhao, Y. et al. Mechanistic analysis of multiple processes controlling solar-driven H_2O_2 synthesis using engineered polymeric carbon nitride. *Nat. Commun.* **12**, 3701 (2021).
3. Du, R. et al. Controlled oxygen doping in highly dispersed Ni-loaded g-C_3N_4 nanotubes for efficient photocatalytic H_2O_2 production. *Chem. Eng. J.* **441**, 135999 (2022).
4. Zhang, P. et al. Heteroatom dopants promote two-electron O_2 reduction for photocatalytic production of H_2O_2 on polymeric carbon nitride. *Angew. Chem. Int. Ed.* **59**, 16209-16217 (2020).
5. Chen, L. et al. Simultaneously tuning band structure and oxygen reduction pathway toward high-efficient photocatalytic hydrogen peroxide production using cyano-rich graphitic carbon nitride. *Adv. Funct. Mater.* **31**, 2105731 (2021).
6. Zhi, Q. et al. Piperazine-linked metalphthalocyanine frameworks for highly efficient visible-light-driven H_2O_2 photosynthesis. *J. Am. Chem. Soc.* **144**, 21328-21336 (2022).
7. Yang, Q., Li, R., Wei, S. & Yang, R. Schottky functionalized Z-scheme heterojunction photocatalyst Ti_2C_3/g-C_3N_4/BiOCl: Efficient photocatalytic

H_2O_2 production via two-channel pathway. *Appl. Surf. Sci.* **572**, 151525 (2022).

8. Jiang, Z. et al. S-scheme ZnO/WO_3 heterojunction photocatalyst for efficient H_2O_2 production. *J. Mater. Sci. Technol.* **124**, 193-201 (2022).

9. Yang, Y. et al. In-situ grown N, S co-doped graphene on TiO_2 fiber for artificial photosynthesis of H_2O_2 and mechanism study. *Appl. Catal. B Environ.* **317**, 121788 (2022).

10. Luo, J. et al. Photoredox-promoted Co-production of dihydroisoquinoline and H_2O_2 over defective $Zn_3In_2S_6$. *Adv. Mater.* **35**, 2210110 (2023).

11. Tsukamoto, D. et al. Photocatalytic H_2O_2 production from ethanol/O_2 system using TiO_2 loaded with Au-Ag bimetallic alloy nanoparticles. *ACS Catal.* **2**, 599-603 (2012).

12. Wang, K. et al. $BiVO_4$ Microparticles decorated with Cu@Au core-shell nanostructures for photocatalytic H_2O_2 production. *ACS Appl. Nano Mater.* **4**, 13158-13166 (2021).

13. Shi, H. et al. Selective modification of ultra-thin g-C_3N_4 nanosheets on the (110) facet of Au/$BiVO_4$ for boosting photocatalytic H_2O_2 production. *Appl. Catal. B Environ.* **297**, 120414 (2021).

14. Shi, H. et al. Mass-transfer control for selective deposition of well-dispersed AuPd cocatalysts to boost photocatalytic H_2O_2 production of $BiVO_4$. *Chem. Eng. J.* **443**, 136429 (2022).

15. Zuo, G. et al. Finely dispersed Au nanoparticles on graphitic carbon nitride as highly active photocatalyst for hydrogen peroxide production. *Catal. Commun.*

123, 69-72 (2019).

16. Wang, Y. et al. Efficient production of H_2O_2 on Au/WO_3 under visible light and the influencing factors. *Appl. Catal. B Environ.* **284**, 119691 (2021).

17. An, R. et al. Decoration of Au NPs on hollow structured BiOBr with surface oxygen vacancies for enhanced visible light photocatalytic H_2O_2 evolution. *J. Solid State Chem.* **306**, 122722 (2022).
